# Clinical Associations of Human T-Lymphotropic Virus Type 1 Infection in an Indigenous Australian Population

**DOI:** 10.1371/journal.pntd.0002643

**Published:** 2014-01-16

**Authors:** Lloyd Einsiedel, Tim Spelman, Emma Goeman, Olivier Cassar, Mick Arundell, Antoine Gessain

**Affiliations:** 1 Flinders University/Northern Territory Rural Clinical School, Alice Springs Hospital, Alice Springs, Northern Territory, Australia; 2 SAPathology, Flinders Medical Centre, Bedford Park, Adelaide, South Australia, Australia; 3 Remote Health, Alice Springs, Northern Territory, Australia; 4 Institut Pasteur, Unité d'Epidémiologie et Physiopathologie des Virus Oncogènes, Département de Virologie, Paris, France; 5 CNRS, UMR 3569, Paris, France; 6 Clinical Information Analyst, Central Australian Health Network, Alice Springs Hospital, Alice Springs, Northern Territory, Australia; George Mason University, United States of America

## Abstract

**Introduction:**

In resource-poor areas, infectious diseases may be important causes of morbidity among individuals infected with the Human T-Lymphotropic Virus type 1 (HTLV-1). We report the clinical associations of HTLV-1 infection among socially disadvantaged Indigenous adults in central Australia.

**Methodology and Principal Findings:**

HTLV-1 serological results for Indigenous adults admitted 1^st^ January 2000 to 31^st^ December 2010 were obtained from the Alice Springs Hospital pathology database. Infections, comorbid conditions and HTLV-1 related diseases were identified using ICD-10 AM discharge morbidity codes. Relevant pathology and imaging results were reviewed. Disease associations, admission rates and risk factors for death were compared according to HTLV-1 serostatus. HTLV-1 western blots were positive for 531 (33.3%) of 1595 Indigenous adults tested. Clinical associations of HTLV-1 infection included bronchiectasis (adjusted Risk Ratio, 1.35; 95% CI, 1.14–1.60), blood stream infections (BSI) with enteric organisms (aRR, 1.36; 95% CI, 1.05–1.77) and admission with strongyloidiasis (aRR 1.38; 95% CI, 1.16–1.64). After adjusting for covariates, HTLV-1 infection remained associated with increased numbers of BSI episodes (adjusted negative binomial regression, coefficient, 0.21; 95% CI, 0.02–0.41) and increased admission numbers with strongyloidiasis (coefficient, 0.563; 95% CI, 0.17–0.95) and respiratory conditions including asthma (coefficient, 0.99; 95% CI, 0.27–1.7), lower respiratory tract infections (coefficient, 0.19; 95% CI, 0.04–0.34) and bronchiectasis (coefficient, 0.60; 95% CI, 0.02–1.18). Two patients were admitted with adult T-cell Leukemia/Lymphoma, four with probable HTLV-1 associated myelopathy and another with infective dermatitis. Independent predictors of mortality included BSI with enteric organisms (aRR 1.78; 95% CI, 1.15–2.74) and bronchiectasis (aRR 2.07; 95% CI, 1.45–2.98).

**Conclusion:**

HTLV-1 infection contributes to morbidity among socially disadvantaged Indigenous adults in central Australia. This is largely due to an increased risk of other infections and respiratory disease. The spectrum of HTLV-1 related diseases may vary according to the social circumstances of the affected population.

## Author Summary

The Human T-Lymphotropic Virus type 1 (HTLV-1) infects at least 5–10 million people worldwide. In developed countries, the most frequently reported HTLV-1 associated diseases include a fatal hematological malignancy, Adult T-cell Leukemia/Lymphoma (ATLL), and the neurological disorder, HTLV-1 associated myelopathy (HAM), which arise in <10% of HTLV-1 carriers during their lifetime. However, most HTLV-1 carriers live in resource-poor areas where infectious diseases, such as strongyloidiasis, could be more important causes of morbidity. Demonstrating such an effect is difficult due to the resource constraints experienced by developing countries in which populations with a substantial burden of infectious diseases reside in areas that are highly endemic for HTLV-1. This is not the case in HTLV-1 endemic central Australia where Indigenous Australians have, for example, among the highest reported blood stream infection rates worldwide in a setting in which sophisticated medical facilities are readily available. We report that bronchiectasis, blood stream infections and admissions with lower respiratory tract infections and strongyloidiasis are associated with HTLV-1 infection. These conditions were far more common than HTLV-1 associated malignancies or neurological conditions in this socially disadvantaged Indigenous population. The spectrum of HTLV-1 related diseases therefore varies according to the social circumstances of the affected population.

## Introduction

The Human T Lymphotropic Virus type 1 (HTLV-1) is an oncogenic retrovirus that preferentially infects CD4+ T cells [1]. Worldwide, HTLV-1 infects at least 5–10 million people who predominantly dwell in areas of high endemicity in southern Japan, the Caribbean basin, parts of South America and inter-tropical Africa. A smaller endemic focus is present in central Australia [2] and we have recently shown this to be due to infection with the HTLV-1c subtype [3]. Epidemiological and clinical associations have been best described for populations in the Caribbean basin, South America and Japan [1]. A minority of HTLV-1 carriers experience clinically significant sequelae, including a rapidly progressive hematological malignancy, Adult T cell Leukemia/Lymphoma (ATLL) [4], [5], and inflammatory disorders, such as HTLV-1 associated myelopathy/tropical spastic paraparesis (HAM/TSP) [6]. A severe exudative eczema, infective dermatitis, predominantly affects children [7]. In Japan and the Caribbean, life-time risks range between 0.3–4% for HAM/TSP, 1–5% for ATL [1] and approach 10% for HTLV-1 associated malignancy or inflammatory diseases overall [1].

Infectious diseases also contribute to HTLV-1 related morbidity and mortality. Severe scabies [8], mycobacterial infections [9] and symptomatic infection with the nematode parasite *Strongyloides stercoralis* [10], [11] are all more frequent among HTLV-1 carriers. In areas endemic for HTLV-1 and *S.stercoralis*, HTLV-1 infection is the major risk factor for complicated strongyloidiasis or ‘hyperinfection’, which is associated with pulmonary involvement [12] and life-threatening sepsis due to enteric bacterial pathogens [13]. Infection with *S.stercoralis* may also reduce the latent period required for the development of ATLL [14]. HTLV-1 infection reduces clearance rates of hepatitis C virus and increases the risk of liver disease and liver disease-related deaths [15]. Whether the risk of chronic hepatitis B virus (HBV) infection is similarly affected is unknown. Interactions between HTLV-1 related inflammatory diseases and infection have also been demonstrated. Infective dermatitis, for example, typically affects HTLV-1 carriers from lower socio-economic backgrounds and predisposes to skin infections with bacterial pathogens [7], which may progress to life-threatening invasive disease [16]. Recently, we reported high rates of HTLV-1 infection among socially disadvantaged Indigenous adults with bronchiectasis in central Australia [17]. Clinically significant pulmonary disease is not a feature of HTLV-1 infection in other developed countries [18]–[20], and we suggested that recurrent lower respiratory tract infections (LRTI) might contribute to this risk in our study population. The spectrum of HTLV-1 related clinical diseases may therefore differ according to social status and the risk of environmental exposure to other pathogens. However, demonstrating such an effect requires diagnostic capabilities that may not be available in developing countries in which a heavy burden of infectious diseases affects a population with a high prevalence of HTLV-1 infection.

**Figure 1:**
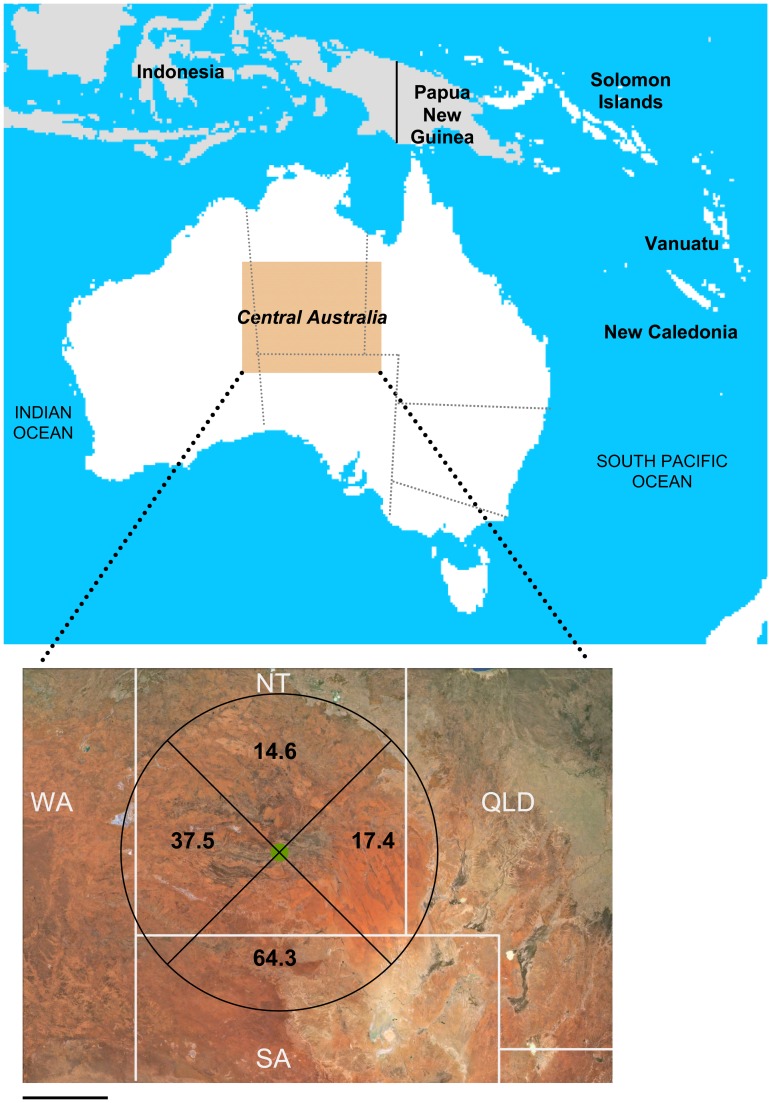
Map of the study area in central Australia including HTLV-1 seropositivity rates for 952 Indigenous adult residents of remote communities divided by quadrant according to their residence relative to the regional center of Alice Springs (green circle). Number of residents tested: North, 335; East, 69; South, 241; West, 307. Abbreviations: NT, Northern Territory of Australia; QLD, Queensland; SA, South Australia; WA, West Australia. Scale bar = 250 km.

Central Australia is well placed to study the associations between poverty and infectious diseases [21]. HTLV-1 is endemic to this region and infects 7.2–13.9% [22], [23] of socially disadvantaged Indigenous adults. There has been no attempt to control HTLV-1 transmission among the Indigenous residents of central Australia, most of whom reside in isolated remote communities in conditions of considerable socio-economic disadvantage [21]. Those who live in the major regional center of Alice Springs dwell in either overcrowded ‘town camps’, which have poor amenities and limited refuse disposal, or are integrated with the majority of the non-Indigenous population within the township's suburbs [21]. Central Australia also has the highest reported blood stream infection (BSI) incidence rates [21] and the highest prevalence rate of adult bronchiectasis [17] worldwide. Prevalence rates of chronic HBV infection exceeded 20% in some communities prior to the introduction of vaccination [24]. Consequently, infection-related mortality rates approach those of some African countries prior to the current HIV pandemic [25]. A single well-resourced community-based hospital, Alice Springs Hospital (ASH), serves this region of 1,000,000 km^2^ (Fig. 1). Critically ill patients are retrieved by air to tertiary referral centers 1,500 km away. Medical services are provided without charge and, notwithstanding the poor social circumstances of the resident population, sophisticated radiological, microbiological and other diagnostic facilities are readily available. The present study describes the spectrum of HTLV-1 associated diseases that affects socially disadvantaged Indigenous adults in central Australia.

## Methods

### Ethics statement

This study was approved by the Central Australian Human Research Ethics Committee, which is a regional committee supervised by the National Health and Medical Research Council of Australia.

### Data collection

All adults (age ≥15 years) admitted to ASH between 1^st^ January 2000 and 31^st^ December 2010 who had an HTLV-1 screening test performed were identified from the hospital pathology data-base. HTLV-1 testing at ASH is performed where there are clinical suspicions of HTLV-1 related diseases, including malignancy, neurological disease, strongyloidiasis and bronchiectasis. Demographic data including ethnicity, dates of birth and death, indigenous status and place of residence were obtained for all patients from the ASH patient management system. For each admission between 1^st^ January 2005 and 31^st^ December 2010 International Statistical Classification of Diseases and Related Health Problems, Tenth Revision, Australian Modification (ICD-10 AM) morbidity codes relating to non-communicable diseases, possible HTLV-1 related conditions and infectious diseases were also extracted (Table S1). Discharge morbidity codes for admissions prior to 2005 were not available and patients who died prior to this date were therefore excluded from statistical analysis. All data were de-identified prior to analysis. Infectious diseases were grouped according to ICD-10 AM codes; i) sepsis or bacterial infection for which a focus was not stated, ii) specified foci of infection and iii) strongyloidiasis (Table S1). HTLV-1 related conditions included ATLL, HAM/TSP, bronchiectasis and infective dermatitis. Cases of ATLL and HAM/TSP were also sought from specialist neurological and hematological units that provide tertiary level care to ASH patients. Case notes, microbiology, radiology and other relevant pathology results were reviewed for all patients with possible HTLV-1 related conditions including ATLL, neurological disorders, bronchiectasis and infective dermatitis.

### Residence

Place of residence was categorized as i) remote (>80 km from Alice Springs), ii) Alice Springs town camp and iii) urban (resident in Alice Springs, but not in a town camp). Remote residence was further divided into quadrants (north, south, east and west) relative to Alice Springs.

### Infections and definitions

Results for *S.stercoralis* serology, HBV serology and blood cultures were obtained from the ASH pathology data-base. During the study period, *S.stercoralis* serology was performed using an in-house enzyme-linked immunosorbent assay based on antigen extracts of *Strongyloides ratti*, which is highly sensitive and specific. A blood culture from which a pathogen was isolated defined a ‘BSI episode’. Repeated culture of the same organism from blood culture was regarded as a separate ‘episode’ only if blood samples were drawn more than one month apart. Blood stream infections excluded potential contaminants including coagulase negative staphylococci, bacillus spp., coryneforms and viridans streptococci unless grown from more than one BC in a 24 hour period and *Acinetobacter spp* in the absence of an identifiable focus. For statistical analysis, the major BSI pathogens were grouped according to their most likely origin: i) skin (*Staphylococcus aureus* and *Streptococcus pyogenes*), ii) respiratory (*Streptococcus pneumoniae* and *Haemophilus influenzae*), iii) urinary tract (*Escherichia coli*) and iv) gastrointestinal tract (Enterobacteriaceae other than *E.*coli). ‘Definite bronchiectasis’ was defined as an ICD-10 AM code for bronchiectasis that was confirmed by High Resolution Computed Tomography (HRCT) chest according to American College of Chest Physicians criteria. ‘Possible bronchiectasis’ was defined as an ICD-10 AM code for bronchiectasis in the absence of HRCT chest confirmation of this diagnosis. A diagnosis of ATLL [4] and HAM/TSP [26] was made using established criteria. Cases of HAM/TSP were categorized as ‘probable’ if the clinical presentation was consistent with HAM/TSP in the absence of confirmatory analysis of cerebrospinal fluid (CSF) [26].

### HTLV-1 studies

Initial screening tests were performed using the Serodia HTLV-1 particle agglutination assay (Fujirebio, Japan) or Architect rHTLV-I/II assay at the Royal Darwin Hospital, Northern Territory of Australia, (1458) or the Institut Pasteur, Paris (156). Positive samples were again tested using both the Serodia HTLV-1 particle agglutination assay and Murex HTLV-I+II test kit (Murex Diagnostics, Dartford, UK)(National Serological Reference Laboratory, Melbourne) or an indirect immunofluorescence assay (IFA) using an HTLV-1-transformed human T cell line (MT2)(Institut Pasteur). HTLV-1 serostatus was then confirmed by Western blot (HTLV Blot 2.4, MP Diagnostics) using stringent criteria for all samples for which screening tests were positive.

### Statistics

Categorical variables were summarized using frequency and percentage and compared using a Chi-square test or, in the case of small numbers, a Fisher's Exact test. Multiple simultaneous comparisons were adjusted for using a Bonferroni correction. Continuous variables were assessed for significant departures from normality with normally distributed variables summarized using mean and standard deviation (SD) and compared using a t-test whilst skewed variables were summarized using median and inter-quartile range (IQR) and compared using a Wilcoxon rank-sum test.

Predictors of HTLV-1 seropositivity were examined using Poisson regression with robust standard errors. Strongyloides admissions (identified by ICD-10 AM codes), rather than serology, were included in the multivariable model because these are more likely to reflect symptomatic strongyloidiasis [10], [11], [27]. Direct modeling of relative risk (RR) using Poisson was preferred over Odds Ratios (OR) from logistic regression to estimate RR due to the frequency of the outcome studied. A link test was used to assess the model for specification error whilst overall goodness of fit was assessed using both visual examination of residuals coupled with a likelihood-ratio test and a Pearson goodness-of-fit test.

Incidence rates of admission count by diagnostic group were expressed as a proportion of the total number of HTLV-1 seropositive and seronegative patients respectively. Predictors of admission counts for a range of diagnostic groups according to HTLV-1 seropositivity were examined using negative binomial regression and are presented with their negative binomial 95% confidence intervals. Negative binomial modeling was preferred over straight Poisson regression due to over-dispersion in admission count outcome variables. The model coefficients represent the estimated change in admission counts for a particular level of a predictor variable. The influence of HTLV-1 seropositivity on admission count was adjusted for demography and comorbidities. In the case of admissions with asthma, LRTI, pneumonia and chronic obstructive pulmonary disease, the model was adjusted for both definite and possible bronchiectasis and tobacco smoking. A link test was used to assess the model for specification error whilst overall goodness of fit was assessed using both visual examination of residuals coupled with a likelihood-ratio test and a Pearson goodness-of-fit test.

Predictors of hepatitis B surface antigen (HBsAg) positivity were analysed using logistic regression. In this case, a logistic approach was preferred secondary to the rarity of the outcome. Overall model fit was assessed using a Hosmer & Lemeshow goodness-of-fit test.

Predictors of time to mortality were examined using Cox Proportional Hazards Regression. Analysis of scaled Schoenfeld residuals were used to assess compliance with the proportional hazards assumption. For this analysis patients with possible bronchiectasis were assumed not to have the condition.

All reported p-values are two-tailed and for each analysis p<0.05 was considered significant. All analyses were conducted using Stata version 12 (StataCorp, College Station, Texas).

## Results

HTLV-1 screening tests were performed for 1614 Indigenous adults and these were positive for 624 (38.7%) cases. Samples from 605 patients were referred for confirmatory Western blot tests. These were indeterminate in 73 cases (4.6%) and confirmed HTLV-1 infection for 531 patients (33.3%). Patients whose western blot results were indeterminate were excluded from further analysis, as were 74 patients (HTLV-1 seropositive, 24; HTLV-1 seronegative, 50) who died prior to 2005. The subsequent analysis therefore included 1451 Indigenous adults (HTLV-1 seropositive, 507; HTLV-1 seronegative, 944) who were admitted 115,919 times (HTLV-1 seropositive 39,967; HTLV-1 seronegative 75,952) during the study period.

### Demographics

HTLV-1 seropositivity rates among males increased significantly with age (<45 years, 106/329 (32.2%); ≥45 years, 135/319 (42.2%); p = 0.008). Rates were otherwise not significantly different between age groups or genders (Table 1). Seropositivity rates differed according to place and type of residence. Rates were lowest among residents of communities north of Alice Springs (14.6%) and highest among those from communities to the south (64.3%) and west (37.5%)(Fig. 1)(Table 1). Seropositivity rates were higher among town camp residents (42.6%) and lowest among those living elsewhere in the township (27.0%). Demographic risk factors for HTLV-1 infection after multivariable analysis included age (adjusted RR, 1.01 per year; 95% CI, 1.01–1.02; p = 0.000) and residence in communities to the south (aRR 3.83; 95% CI, 2.64–5.57; p = 0.000) and west (aRR 2.77; 95% CI, 1.54–3.37; p = <0.001) of Alice Springs relative to those in the north (Table 2).

**Table 1:** Patient characteristics for 1451 Indigenous Adults admitted 2005–2010^a^.

		HTLV-1 WB result		
	Level	Positive (n = 507)	Negative (n = 944)	p-value
Sex n (% of level)	Male/Female	241 (45.4)/266 (50.1)	407 (41.0)/537 (54.0)	0.106
Age at test, median years (IQR)		47.1 (38.7, 57.4)	43.5 (32.9,55.3)	<0.001
Age				
<45 years	Male/Female	106 (32.2)/116 (29.9)	223 (67.7)/272 (70.1)	0.503^b^
≥45 years	Male/Female	135 (42.2)/149 (36.1)	184 (57.8)/264 (63.9)	0.086^b^
Residence^c^, n (% of level)				
	Town Camp	107 (42.6)	144 (57.4)	0.001^g^
	Remote	308 (38.5)	493 (61.6)	
	Nursing Home	18 (34.6)	34 (65.4)	
	Urban	57 (27.0)	154 (73.0)	
Quadrant^d^, n (% of level)				
	North (n = 335)	49 (14.6)	286 (85.4)	<0.001^f^
	East (n = 69)	12 (17.4)	57 (82.6)	
	South (n = 241)	155 (64.3)	86 (35.7)	
	West (n = 307)	115 (37.5)	192 (62.5)	
Death^e^, n (%)		120 (23.7)	218 (23.1)	0.805
Age at death, median years (IQR)		56.9 (46.2,63.9)	53.2 (44.4,62.5)	0.235

^a^ Excluding patients with an indeterminate western blot and those who died prior to 2005.

^b^ Analyzed according to gender within each group.

^c^ Excluding 134 patients who resided outside central Australia and 2 patients whose place of residence was unknown. Data are expressed as proportion of total patients tested for each place of residence.

^d^ Residents of remote communities relative to the regional center of Alice Springs and excluding 498 Alice Springs residents. Data are expressed as proportion of total patients tested for each quadrant.

^e^ Died during observation period.

^f^ All pair-wise quadrant comparisons were p<0.001 (Bonferroni-corrected), except the North vs East comparison.

^g^ Pair-wise comparisons of urban-residence compared with all other residences were p<0.001 (Bonferroni-corrected).

**Table 2:** Adjusted Poisson modeling of predictors of HTLV-1 infection.

	Relative Risk	95% CI	p-value
Age	1.01	1.01–1.02	0.000
Male Gender	1.00	0.88–1.15	0.968
Residence			
Type			
Remote	reference		
Town Camp	0.940	0.57–1.55	0.809
Urban	0.711	0.42–1.21	0.211
Remote Area^a^			
North	reference		
East	1.34	0.73–2.44	0.347
South	3.83	2.64–5.57	<0.001
West	2.77	1.54–3.37	<0.001
Bronchiectasis^b^	1.35	1.14–1.60	0.001
ICD-10 AM coded conditions^c^			
Alcohol	1.45	1.25–1.68	0.000
Malignancy^d^	0.51	0.32–0.83	0.007
Strongyloides^e^	1.38	1.16–1.64	0.000
Microbiology			
BSI^f^			
Enteric	1.36	1.05–1.77	0.020
Skin	1.14	0.94–1.39	0.187
Respiratory	1.19	0.95–1.48	0.128
HBsAg	1.18	0.98–1.42	0.089

^a^ Residence in remote communities relative to the regional center of Alice Springs.

^b^ Patients for whom an ICD-10 AM discharge morbidity code of bronchiectasis was recorded and where this was confirmed by HRCT.

^c^ Conditions identified from discharge morbidity codes.

^d^ Excluding hematological malignancies.

^e^ Strongyloides identified by ICD-10 AM code.

^f^ Blood stream infections identified from blood cultures. Enteric pathogens, Enterobacteriaceae other than *Escherichia coli*; skin pathogens, *Staphylococcus aureas* and *Streptococcus pyogenes*; respiratory pathogens, *Streptococcus pneumoniae* and *Haemophilus influenzae*.

Abbreviations: BSI, blood stream infection; HBsAg, Hepatitis B surface antigen positive.

### Medical conditions previously associated with HTLV-1 infection

#### i) Respiratory diseases

A bronchiectasis-related discharge morbidity code was recorded for 170 patients. Bronchiectasis was confirmed radiologically in 142 patients of whom 81 (57.0%) were HTLV-1 seropositive. Radiologically confirmed bronchiectasis was an independent predictor of HTLV-1 infection in a multivariable model (aRR 1.35; 95%CI, 1.14–1.60; p = 0.001) (Table 2). HTLV-1 carriers were also more likely to be admitted with LRTI other than pneumonia, pneumonia and bronchiectasis (Table 3) and had higher admission numbers for asthma, LRTI other than pneumonia, pneumonia and bronchiectasis (Table 4). These associations remained after adjusting for demographic factors and comorbidities (Table 5).

**Table 3:** Comparison of clinical conditions identified by International Classification of Diseases-10 (Australian Modification) morbidity codes according to HTLV-1 serostatus among 1451 Indigenous adults admitted 2005–2010^a^.

	HTLV-1 Positive (n = 507) n (%)	HTLV-1 Negative (n = 944) n (%)	p-value
Non-Communicable Diseases			
Smoking	316 (62.3)	538 (57.0)	0.049
Alcohol	275 (54.2)	380 (40.3)	<0.001
Diabetes	252 (49.7)	467 (49.5)	0.932
CKD	164 (32.3)	297 (31.5)	0.730
HD	107 (21.1)	214 (22.7)	0.600
CCF	94 (18.5)	144 (15.3)	0.107
CLD	68 (13.4)	88 (9.3)	0.016
Malignancy^b^	12 (2.4)	49 (5.2)	0.011
Infections			
Sepsis/No focus	391 (77.1)	621 (65.8)	<0.001
Pneumonia	316 (62.3)	477 (50.5)	<0.001
LRTI^c^	227 (47.2)	342 (40.0)	0.002
Skin	207 (40.8)	349 (37.0)	0.159
Scabies	72 (14.2)	80 (8.5)	0.001
Bone/Joint	51 (10.1)	92 (9.8)	0.849
Respiratory Diseases			
Bronchiectasis^d^	81 (16.8)	61 (7.1)	<0.001
COPD	78 (15.4)	82 (8.7)	<0.001
Asthma	34 (7.1)	54 (6.3)	0.46
HTLV-1 associated conditions			
*Strongyloides stercoralis* e	111 (23.1)	157 (18.3)	0.039
HAM/TSP^f^	4 (0.8)	0	**
ATLL	2 (0.4)	0	**
Infective dermatitis	1 (0.2)	0	**

Data derived from 115,919 admissions (HTLV-1 seropositive 39,967; HTLV-1 seronegative, 75,952) among 481 HTLV-1 seropositive and 856 HTLV-1 seronegative Indigenous adults.

^a^ Excluding patients with an indeterminate western blot and those who died prior to 2005.

^b^ Non-hematological malignancies.

^c^ LRTI other than pneumonia.

^d^ Bronchiectasis confirmed by chest high resolution computed tomography.

^e^ Identified by ICD-10 AM coding.

^f^ Probable HAM/TSP. Confirmatory tests not applied to cerebrospinal fluid.

no p-value provided due to small numbers recorded.

Abbreviations: ATLL, adult T cell leukemia/lymphoma; CCF, congestive cardiac failure; CKD, chronic kidney disease; CLD, chronic liver disease; HAM/TSP, HTLV-1 associated myelopathy/tropical spastic paraparesis; HD, hemodialysis; LRTI, lower respiratory tract infection; WB, Western blot.

**Table 4:** Admission rates for respiratory conditions and other infections according to HTLV-1 serostatus.

Category	HTLV-1 positive (n = 490)	HTLV-1 negative (n = 827)	p-value
	(admissions/patient)	(admissions/patient)	
Respiratory Diseases^a^			
Asthma	0.67	0.19	<0.0001
LRTI^b^	1.33	0.86	<0.0001
Pneumonia	2.05	1.32	<0.0001
Bronchiectasis	1.95	0.87	<0.0001
COPD	0.48	0.43	0.1872
Infections^c^			
Sepsis	3.98	3.07	<0.0001
BSI episodes^d^	0.58	0.42	0.0001
Strongyloides	0.23	0.11	<0.0001
Scabies	0.19	0.14	0.0385

Admission rates for 1317 adult Indigenous residents of central Australia 2005–2010 admitted to Alice Springs Hospital with respiratory conditions and infections. Excluding patients who died prior to 2005 and those residing outside central Australia for whom admission data was incomplete.

^a^ Identified by ICD-10 AM code. Bronchiectasis was confirmed by chest high resolution computed tomography.

^b^ LRTI other than pneumonia.

^c^ Identified by ICD-10 AM coding with the exception of BSI episodes.

^d^ The number of blood cultures that yielded a significant pathogen as defined in methods.

Abbreviations: BSI, blood stream infection; COPD, chronic obstructive pulmonary disease; LRTI, lower respiratory tract infection.

**Table 5:** Adjusted negative binomial regression of predictors for number of admissions^a^ with respiratory conditions and other infections according to HTLV-1 serostatus.

Category	Coefficient^b^	95% CI	p-value
Respiratory Diseases^c^			
Asthma	0.986	0.271, 1.701	0.007
LRTI^d^	0.254	0.067, 0.441	0.008
Pneumonia	0.189	0.039, 0.340	0.014
Bronchiectasis	0.598	0.015, 1.180	0.044
COPD	0.214	−0.257, 0.685	0.374
Infections^e^			
Sepsis	0.123	−0.017, 0.264	0.085
BSI episodes^f^	0.210	0.016, 0.405	0.034
Strongyloides	0.563	0.174, 0.953	0.005
Scabies	0.358	−0.011, 0.726	0.057

Adjusted negative binomial modeling of predictors for admission to Alice Springs Hospital among 1317 adult Indigenous residents of central Australia, 2005–2010. Excluding patients who died prior to 2005 and those residing outside central Australia for whom admission data was incomplete.

^a^ Adjusted for comorbidities (harmful alcohol consumption, diabetes, chronic liver disease, chronic kidney disease, hemodialysis), age, gender and place of residence. Respiratory conditions were also adjusted for smoking and, in the case of asthma, LRTI and pneumonia, for definite or possible bronchiectasis.

^b^ The coefficient represents the average change in the number of admissions that is associated with the presence of the predictor variable according to HTLV-1 serostatus.

^c^ Identified by ICD-10 AM code. Bronchiectasis was confirmed by high resolution computed tomography chest.

^d^ LRTI other than pneumonia.

^e^ Identified by ICD-10 AM code with the exception of BSI episodes.

^f^ The number of blood cultures that yielded a significant pathogen as defined in methods.

Abbreviations: BSI, blood stream infection; COPD, chronic obstructive pulmonary disease; LRTI, lower respiratory tract infection.

#### ii) Strongyloidiasis

Strongyloides serology was performed for 1126 (77.6%) patients of whom 269 (23.9%) were Strongyloides seropositive (Table 6). Although HTLV-1 carriers were more likely to record a Strongyloides serology result (Table 6), Strongyloides seropositivity rates were not significantly higher in this group (HTLV-1 seropositive, 27.1%; HTLV-1 seronegative, 22.0% (p = 0.063)(Table 6). Routine stool microscopy was performed in only 47 cases at the time of diagnosis with strongyloidiasis and stool was cultured for strongyloides in only eight of these cases. Larvae were identified in 19 cases (HTLV-1 seronegative, 7; HTLV-1 seropositive, 12). The numbers of admissions with strongyloidiasis were significantly higher among HTLV-1 carriers (Table 4) and the likelihood of admission with strongyloidiasis remained increased in a multivariable model (aRR 1.38; 95% CI, 1.16–1.64; p = 0.000) (Table 2).

**Table 6:** Results of microbiological tests for 1451 Indigenous Adults admitted 2005–2010^a^.

	HTLV-1 WB result		
	Positive (n = 507) n (%)	Negative (n = 944) n (%)	p-value
Blood Stream Infections	181 (35.7)	254 (26.9)	<0.001
Strongyloides serology			
Tested	409 (80.7)	717 (76.0)	0.040
Positive	111 (27.1)	158 (22.0)	0.063^a^
Borderline	61 (14.9)	115 (16.0)	
Negative	237 (58.0)	444 (61.9)	
Hepatitis B Virus serology			
Tested	337 (66.5)	651 (69.0)	0.290
Anti-HBc	201 (59.6)	338 (51.9)	0.021
HBsAg	65 (32.3)	62 (18.3)	<0.001
HBeAg	5 (7.7)	11 (17.7)	0.077

^a^ Pair-wise comparisons of Strongyloides serological results were Bonferroni-corrected.

Abbreviations: HBV, hepatitis B virus; anti-HBc, hepatitis B core antibody positive; HBeAg, hepatitis B e antigen positive; HBsAg, hepatitis B surface antigen positive; WB, Western blot.

#### iii) Scabies

HTLV-1 carriers were more likely to record a discharge morbidity code for scabies (Table 3) and had higher admission rates for this condition (Table 4); however, this association was lost in an adjusted model (Table 5). Severity of scabies could not be determined from ICD-10 AM codes and skin scrapings were performed for few patients.

#### iv) Malignancy, HAM/TSP and infective dermatitis

The risk of non-hematological malignancies was significantly reduced among HTLV-1 carriers (Table 2). Six patients were admitted with hematological malignancies including two who were diagnosed with ATLL after referral to a tertiary hospital. The clinical presentation of four HTLV-1 seropositive patients was consistent with HAM/TSP; however, in no case were HTLV-1 specific investigations applied to CSF. A single patient with HTLV-1 related infective dermatitis was identified.

### Bacterial infections

Nearly 70% of patients (HTLV-1 seropositive, 391; HTLV-1 seronegative, 621) recorded at least one discharge code for sepsis with no focus specified during the study period. Although HTLV-1 carriers more often recorded discharge codes for sepsis with no focus specified (Table 3) and had higher admission numbers for this category (Table 4), these associations were lost after adjusting for covariates (Table 5). HTLV-1 carriers were also more likely to experience a BSI (Table 6) and had more BSI episodes after adjusting for covariates (Table 5). When analyzed according to the most likely origin of infection, BSI from a probable gastrointestinal source remained significantly associated with HTLV-1 infection in a multivariable model (aRR, 1.36; 95% CI, 1.05–1.77; p = 0.020) (Table 2).

### Hepatitis B Virus

Among 988 (68.1%) patients tested, 127 (12.9%) were HBsAg positive of whom 16 (12.6%) were also HBeAg positive (Table 6). The geographic distribution of HBsAg positivity was similar to that of HTLV-1 seropositivity. Risk was greatest among residents of remote communities to the south (unadjusted odds ratio (uOR), 3.98; 95% CI, 2.23–7.10) and west (uOR, 2.23; 95% CI, 1.25–3.99) compared with northern communities and was reduced for urban relative to remote residents (uOR, 0.30; 95% CI, 0.14–0.64). Although HTLV-1 infected patients were more likely to be HBsAg positive (HTLV-1 seropositive, 65/201 (32.3%); HTLV-1 seronegative, 62/338 (18.3%)(p = <0.001) (Table 6), exposure to HBV was more frequent among HTLV-1 seropositive patients (anti-HBc positive: HTLV-1 seropositive, 59.6%; HTLV-1 seronegative, 51.9%)(p = 0.021)(Table 6) and HBsAg positivity was not associated with HTLV-1 infection in a multivariable model (Table 2).

### Mortality

Among 338 deaths that occurred during 5,739 years of follow-up, 120 (23.7%) were HTLV-1 seropositive and 218 (23.1%) were HTLV-1 seronegative. There was no difference between HTLV-1 seropositive and seronegative patients in median age of death (HTLV-1 seropositive, 56.9 years; IQR, 46.2, 63.9); HTLV-1 seronegative, 53.2 years; IQR, 44.4, 62.5) (Table 1). Demographic risk factors for death included male gender and increasing age (Table 7).

**Table 7:** Adjusted Cox proportional hazards modeling of predictors of death^a^.

	Hazard Ratio	95% Confidence Interval	p-value
Age^b^	1.03	1.02–1.04	0.000
Male Gender	1.34	1.07–1.68	0.011
Residence^c^			
Type			
Remote	Reference		
Town Camp	1.11	0.82–1.51	0.506
Urban	1.03	0.58–1.83	0.914
Comorbidities^d^			
Bronchiectasis^e^	2.07	1.45–2.98	0.000
Diabetes	1.45	1.08–1.95	0.013
Chronic Liver Disease	1.91	1.43–2.56	0.000
Chronic Kidney Disease	1.19	0.88–1.62	0.264
Malignancy	1.81	1.21–2.69	0.004
Cardiac Failure	1.29	0.98–1.69	0.070
Infection			
HTLV-1	0.80	0.62–1.03	0.085
Strongyloides^f^	1.11	0.96–1.28	0.169
Blood Stream Infections^g^			
Enterobacteriaceae^h^	1.78	1.15–2.74	0.009
*Klebsiella pneumoniae*	1.06	0.57–1.96	0.849
*Staphylococcus aureus*	0.77	0.28–2.14	0.620
*Streptococcus pneumoniae*	1.70	1.09–2.64	0.018
HBsAg positive	1.10	0.76–1.61	0.605

^a^ Including 338 deaths that occurred after 1^st^ January 2005.

^b^ Risk of death for each 5 years increase in age.

^c^ Excluding 134 patients who resided outside central Australia and 2 patients whose place of residence was unknown.

^d^ Identified by ICD-10 AM coding.

^e^ Definite bronchiectasis identified by ICD-10 AM code and confirmed by High Resolution Computed Tomography.

^f^ Strongyloides identified by ICD-10 AM code.

^g^ Blood stream infections identified from blood cultures.

^h^ Excluding *Escherichia coli*.

Abbreviations: HBsAg, hepatitis B surface antigen; HTLV-1, Human T-Lymphotropic Virus type 1.

Bronchiectasis (HR, 2.07; 95% CI, 1.45–2.98; p = 0.000) and BSI with Enterobacteriaceae other than *E.coli* (HR 1.78; 95% CI, 1.15–2.74; 0.009) remained significant predictors of death after multivariable analysis (Table 7). Other risk factors for death were *S.pneumoniae* BSI (HR, 1.70; 95% CI, 1.09–2.64; p = 0.018) and non-communicable diseases (chronic liver disease, diabetes and malignancy)(Table 7).

## Discussion

In a hospitalized cohort of Indigenous Australian adults, we found an HTLV-1 seropositivity rate (33.3%) that was approximately three times the estimated background rate in central Australia (7.2–13.9%) [22], [23]. This suggests that HTLV-1 associated morbidity in our study population may substantially exceed that resulting from the occasional cases of ATLL and HAM/TSP that are reported here. Consistent with its global epidemiology [2], HTLV-1 carriers were more likely to live in poverty in town camps or remote communities and more often had a history of harmful alcohol consumption. HTLV-1 infection was associated with strongyloidiasis and blood stream infections with enteric pathogens; however, respiratory diseases contributed most to HTLV-1 related morbidity in this socially disadvantaged Indigenous population. After adjusting for covariates, HTLV-1 infection was associated with bronchiectasis and with increased admission numbers for all respiratory conditions studied with the exception of chronic obstructive pulmonary disease.

Pulmonary involvement is common among HTLV-1 carriers elsewhere. Radiological abnormalities, for example, have been reported in 50% of Japanese patients with HAM/TSP and 30% of asymptomatic HTLV-1 carriers who were examined by chest X-ray [28] and chest CT [29], respectively. Airway involvement is frequent in this population; chest CT reveals bronchiolitis or bronchitis in 19% [30] and bronchiectasis in 18–26% [29], [30] of cases. Lymphocyte infiltration of bronchioles [31] and partial bronchiolar obstruction [31], [32] are the histopathological correlates of these radiological findings. Lymphocytes obtained from HTLV-1 carriers by bronchoalveolar lavage (BAL) have high HTLV-1 proviral loads [33], [34] and these are correlated with those of peripheral blood [31]. An inflammatory response to the HTLV-1 antigen load derived from infected lymphocytes is thought to be the major determinant of other HTLV-1 related inflammatory diseases [35]. Airway inflammation in response to HTLV-1 antigens, such as the immuno-dominant regulatory protein, Tax [30], may therefore provide the pathological basis for clinical associations with asthma and LRTI other than pneumonia in our Indigenous cohort and for the increased incidence of self-reported asthma among HTLV-1 carriers in the USA [20].

Nevertheless, clinically significant pulmonary disease is an uncommon feature of HTLV-1 infection in developed countries [18]–[20]. In contrast, HTLV-1 infection contributes to bronchiectasis prevalence rates among Indigenous adults in central Australia that are the highest reported worldwide [17]. In the present study, 142 cases of bronchiectasis were confirmed by HRCT and nearly 60% of these patients were HTLV-1 infected. Consistent with our previous study [17], bronchiectasis was associated with a very high early mortality. Previously we have shown that HTLV-1 infection is associated with more extensive bronchiectasis, more frequent right heart failure and with bronchiectasis-related deaths [17]. In a recent case-control study the mean HTLV-1 proviral load in peripheral blood lymphocytes was significantly higher among HTLV-1 infected patients with bronchiectasis [36]. An HTLV-1 mediated inflammatory process [35] may therefore underlie HTLV-1 associated pulmonary disease in our study population. Disease progression to multifocal bronchiectasis might then follow further pulmonary injury resulting from recurrent LRTI, which were more common among HTLV-1 carriers in the present study.

Consistent with the results of other studies [27], [37], HTLV-1 carriers in central Australia were not at increased risk of serologically defined strongyloidiasis. Nevertheless, HTLV-1 infection in other populations is associated with a higher larval burden and with increased risks of symptomatic, recurrent and complicated strongyloidiasis [10], [11], [27]. Our study design and the use of serological tests to diagnose strongyloidiasis preclude any assessment of disease severity. However, HTLV-1 carriers in the present study were more likely to be admitted with a diagnosis of strongyloidiasis and had higher admission numbers for this condition, findings that could result from a higher larval burden. Unfortunately, our analysis of admission numbers for strongyloidiasis might also be confounded by the acknowledged disease association with HTLV-1 infection, which may lower the clinical threshold for administering antihelminthics to HTLV-1 carriers and increase the likelihood that a Strongyloides-related ICD-10 AM code is recorded. The association between HTLV-1 infection and strongyloidiasis in central Australia therefore requires confirmation in a prospective study. Nevertheless, high rates of *S.stercoralis* infection were found among Indigenous adults in an arid region of Australia that would appear otherwise hostile to soil transmitted helminths. The presence of HTLV-1 infected ‘core transmitters’ who carry a high larval burden may be central to the survival of *S.stercoralis* in this environment and could increase the risk of *S.stercoralis* infection among other community members. Strongyloidiasis may also contribute to the very high BSI incidence rates that have been reported in central Australia [38]. Among Indigenous adults in this region, enteric gram-negative bacilli are the most common pathogens isolated from blood [38] and we have previously reported BSI-related deaths among patients with complicated strongyloidiasis [13].

In our Indigenous Australian cohort, respiratory and infection-related morbidity were increased among HTLV-1 carriers in the absence of an increased risk of death. However, an effect of HTLV-1 infection on mortality may be obscured by analysis according to HTLV-1 serological status rather than HTLV-1 proviral load, which is closely associated with HTLV-1 related diseases [1]. Certainly, the recent finding of higher HTLV-1 proviral loads among HTLV-1 carriers with bronchiectasis [36] suggests that stratifying mortality by HTLV-1 proviral load may more accurately reflect risk in our patient population. Interestingly, an increased risk of death among HTLV-1 carriers in Guinea-Bissau [39], [40] is associated with higher HTLV-1 proviral loads [41]. A modest increase in all-cause mortality has also been reported among HTLV-1 carriers in Japan [42]; however, no such association has been found for blood donors in the USA [43]. These geographic differences in HTLV-1 associated mortality might reflect environmental conditions in resource poor areas that predispose to recurrent respiratory tract infections and expose HTLV-1 carriers to other pathogens, such as *Mycobacterium tuberculosis* [39] and *S.stercoralis* [12].

The retrospective nature of this study results in a number of limitations. First, patients with HTLV-1 related diseases were identified from discharge morbidity codes. Attempts were made to identify other patients with these conditions by contacting specialist medical units to which such patients are referred; however, cases may have been missed if these were not coded or referred appropriately. Consequently, our data are likely to under-estimate the actual burden of HTLV-1 related diseases in this population. The risk of bronchiectasis attributable to HTLV-1 infection is also likely to be underestimated because individuals who had not received radiological confirmation of this diagnosis were assumed not to have the condition. Similarly, the effect of HTLV-1 infection on respiratory conditions may be underestimated because ‘possible’ bronchiectasis was included in the final model to account for the increased risk of respiratory infection resulting from structural lung disease. Determining the strength of other possible associations was dependent on the accuracy of discharge coding; however, with the possible exception of strongyloidiasis noted above, this is unlikely to vary according to HTLV-1 serological status. Finally, we assumed that HTLV-1 infection was acquired in most cases prior to the period in which ICD-10 AM codes were collected. The low annual incidence rate of HTLV-1 seroconversion among discordant couples [1] suggests that this is likely to be the case. Indeed, vertical transmission may be relatively more important in our study population due to the substantial risks posed by the custom of prolonged breast-feeding [44]. Strengths of the study are the large sample size, which included 10% of the region's Indigenous adult resident population, the presence of a single well-resourced hospital that serves this population and the use of data from different sources to study the HTLV-1 related associations reported here.

In a setting of overcrowded housing, inadequate health hardware and poor community hygiene [45], [46], HTLV-1 infection substantially increases respiratory and infection-related morbidity. Socially disadvantaged HTLV-1 carriers in our Indigenous Australian cohort experienced more BSI episodes and were more often admitted with respiratory conditions including LRTI and bronchiectasis, which was the major independent risk factor for death. In contrast to other developed countries [1], infection-related complications were more common than either ATLL or HAM/TSP. The spectrum of HTLV-1 related diseases is therefore likely to vary according to the social circumstances of the affected population. These findings have not been reported previously; however, access to the medical facilities required to confirm these diagnoses is limited in developing countries in which populations with a similar burden of disease exists. Clearly, the benefits accrued by controlling the vertical transmission of HTLV-1 in a resource poor setting must be considered relative to the capacity of the health care system to ensure the safety of alternative sources of infant nutrition. However, our data provides strong support for public health interventions, such as improvements to housing and community hygiene, that limit the exposure of HTLV-1 carriers to other pathogens.

## Supporting Information

Table S1 **Disease categories and their ICD-10 AM codes recorded 2005–2010.** International classification of diseases 10^th^ revision, Australian modification, codes recorded for 1337 Indigenous adults admitted to Alice Springs Hospital, 2005–2010. These codes formed the basis for subsequent analysis according to the categories listed. Abbreviations: COPD, chronic obstructive pulmonary disease; LRTI, lower respiratory tract infection; ICD-10 AM, international classification of diseases 10 Australian Modification.
(DOCX)
