# Supplementary material for: Clinical Associations of Human T-Lymphotropic Virus Type 1 Infection in an Indigenous Australian Population
Source: PLoS Negl Trop Dis. 2014 Jan 16;8(1):e2643. doi: 10.1371/journal.pntd.0002643 (PMC3894183; doi:10.1371/journal.pntd.0002643)
Supplement: Table S1 — Disease categories and their ICD-10 AM codes recorded 2005–2010. International classification of diseases 10th revision, Australian modification, codes recorded for 1337 Indigenous adults admitted to Alice Springs Hospital, 2005–2010. These codes formed the basis for subsequent analysis according to the categories listed. Abbreviations: COPD, chronic obstructive pulmonary disease; LRTI, lower respiratory tract infection; ICD-10 AM, international classification of diseases 10 Australian Modification. (DOCX) [file pntd.0002643.s001.docx]

Table S1: Disease categories and their ICD-10 AM codes recorded 2005-2010.

| Category | ICD 10 AM Codes Recorded |
| --- | --- |
| **Comorbidities** |  |
| Malignancy | C04.9, C06.9, C09.9, C12, C13.9, C15.5, C16.0, C16.3, C17.0, C18.2, C18.9, C20, C22.0, C30.0, C31.0, C34.0, C34.1, C34.3, C34.9, C44.0, C44.1, C44.3, C44.5, C44.6, C44.7, C50.1, C50.4, C50.9, C51.9, C53.9, C54.1, C60.9, C64, C67.9, C71.6, C71.9, C77.0, C77.1, C77.2, C77.3, C77.4, C78.0, C78.1, C78.5, C78.7, C79.5, C79.88, C80 |
| Hematological Malignancies | C83.0, C84.4, C84.5, C91.10, C92.00, C95.90 |
| Diabetes | E11.01, E11.02, E11.11, E11.12, E11.13, E11.21, E11.22, E11.23, E11.29, E11.31, E11.33, E11.34, E11.35, E11.36, E11.39, E11.40, E11.41, E11.42, E11.43, E11.49, E11.51, E11.52, E11.61, E11.62, E11.63, E11.64, E11.65, E11.69, E11.71, E11.72, E11.73, E11.9 |
| Harmful Alcohol Consumption | F10.0, F10.1, F10.2, F10.3, F10.4, F10.5, F10.6, F10.7, F10.8, F10.9 |
| Tobacco Use | F17.1, F17.2, Z72.0 |
| Peripheral Neuropathy | G60.0, G62.9 |
| Spastic Paraparesis | G04.1, G82.10 |
| Epilepsy | G40.10, G40.20, G40.21, G40.30, G40.40, G40.50, G40.51, G40.60, G40.90, G40.91, G41.0, G41.2, G41.9 |
| Coronary Artery Disease | I20.0, I20.1, I20.8, I20.9, I21.0, I21.1, I21.2, I21.3, I21.4, I21.9, I22.9, I23.2, I24.1, I24.9, I25.10, I25.11, I25.2, I25.5, I25.8, I25.9 |
| Cardiac Failure | I42.0, I50.0, I50.1, I50.9, I51.7 |
| Chronic Liver Disease | K70.3 |
| Asthma | J45.0, J45.1, J45.9, J46 |
| Bronchiectasis | J47 |
| COPD | J40, J41.1, J42, J43.8, J43.9, J44.0, J44.1, J44.8, J44.9 |
| Skin Disorders | L21.9, L29.9, L30.3, L30.9, L50.0, L50.1, L50.9, L85.0 |
| Connective Tissue Disorders | L93.0, M32.1, M32.8, M32.9, M33.2, M34.0, M34.1, M34.9, M35.0, M35.1, M35.9, |
| Chronic kidney disease |  |
| Stage 3-Stage 4 | N18.3, N18.4, N18.8, N18.9, N18.90, N18.91 |
| Stage 5 | N18.5, Z49.1, Z49.2 |
| **Infections** |  |
| Strongyloidiasis | B78.0, B78.7, B78.9 |
| Mycobacterial Infections | A15.0, A15.1, A16.0, A16.1, A16.2, A16.9, A18.3, A31.0, A31.8, A31.9 |
| Sepsis/Bacterial infection-no focus identified | A39.2, A40.0, A40.1, A40.2, A40.3, A40.8, A40.9, A41.0, A41.1, A41.2, A41.3, A41.50, A41.51, A41.52, A41.58, A41.8, A41.9, A49.0, A49.1, A49.2 |
| Meningitis | G00.0, G00.1, G00.3, G00.8, G00.9, G02.1, G03.9, G04.2, G04.9, G06.0, G06.1 |
| Endocarditis | I33.0, I38 |
| Pneumonia | J13, J14, J15.0, J15.1, J15.2, J15.4, J15.5, J15.6, J15.7, J15.8, J15.9, J16.8, J17.0, J17.2, J18.0, J18.1, J18.9 |
| LRTI other than pneumonia | J20.1, J20.9, J21.0, J21.8, J21.9, J22 |
| Bacterial Skin Infections | L01.0, L01.1, L02.0, L02.1, L02.2, L02.3, L02.4, L02.8, L03.01, L03.02, L03.10, L03.11, L03.2, L03.3, L03.8, L08.0, L08.8, L08.9 |
| Scabies | B86 |
| Bone and Joint Infections | M00.00, M00.04, M00.06, M00.17, M00.24, M00.26, M00.84, M00.87, M00.91, M00.92, M00.94, M00.95, M00.96, M00.97, M01.30, M01.31, M01.32, M01.35, M01.36, M46.22, M46.24, M86.10, M86.14, M86.16, M86.17, M86.18, M86.43, M86.45, M86.47, M86.64, M86.65, M86.66, M86.67, M86.68, M86.88, M86.91, M86.94, M86.95, M86.96, M86.97, M86.98 |
| Urinary Tract Infections | N39.0 |

Table S1 Legend. International classification of diseases 10^th^ revision, Australian modification, codes recorded for 1337 Indigenous adults admitted to Alice Springs Hospital, 2005-2010. These codes formed the basis for subsequent analysis according to the categories listed.

Abbreviations: COPD, chronic obstructive pulmonary disease; LRTI, lower respiratory tract infection; ICD-10 AM, international classification of diseases 10 Australian Modification.
